# Income and rural–urban status moderate the association between income inequality and life expectancy in US census tracts

**DOI:** 10.1186/s41043-023-00366-6

**Published:** 2023-03-28

**Authors:** Steven A. Cohen, Caitlin C. Nash, Erin N. Byrne, Mary L. Greaney

**Affiliations:** Department of Health Studies, College of Health Sciences, University of Rhode Island, 25 West Independence Way, Suite P, Kingston, RI 02881 USA

**Keywords:** Socioeconomic status, Income inequality, Population health, Geography

## Abstract

**Background:**

A preponderance of evidence suggests that higher income inequality is associated with poorer population health, yet recent research suggests that this association may vary based on other social determinants, such as socioeconomic status (SES) and other geographic factors, such as rural–urban status. The objective of this empirical study was to assess the potential for SES and rural–urban status to moderate the association between income inequality and life expectancy (LE) at the census-tract level.

**Methods:**

Census-tract LE values for 2010–2015 were abstracted from the US Small-area Life Expectancy Estimates Project and linked by census tract to Gini index, a summary measure of income inequality, median household income, and population density for all US census tracts with non-zero populations (*n* = 66,857). Partial correlation and multivariable linear regression modeling was used to examine the association between Gini index and LE using stratification by median household income and interaction terms to assess statistical significance.

**Results:**

In the four lowest quintiles of income in the four most rural quintiles of census tracts, the associations between LE and Gini index were significant and negative (*p* between < 0.001 and 0.021). In contrast, the associations between LE and Gini index were significant and positive for the census tracts in the highest income quintiles, regardless of rural–urban status.

**Conclusion:**

The magnitude and direction of the association between income inequality and population health depend upon area-level income and, to a lesser extent, on rural–urban status. The rationale behind these unexpected findings remains unclear. Further research is needed to understand the mechanisms driving these patterns.

## Introduction

Compared to more equitable societies, those with wider income gaps between rich and poor have worse population health outcomes [1, 2], including obesity [3], cancer [4], cardiovascular disease [5], and mortality [4, 6]. A 2018 longitudinal study found a strong association between state-level income inequality and life expectancy (LE) [7], a commonly used summary measure of population health representing the average number of years a person in an area can expect to live, based on current age-specific mortality rates [8]. The consensus in public health is the more equitable society is, the better the population health [2].

However, empirical evidence suggests that the association between income inequality and population health is more nuanced. For example, research has determined that the strength of these associations between LE and income inequality in the USA was significantly stronger in areas of higher overall income [4] and in rural areas compared to urban areas [6]. Results of a Canadian study further suggest that rural populations may be more vulnerable to the influence of income inequality on health due to certain population and geographic characteristics, such as reduced access to basic health care services and greater socioeconomic and demographic homogeneity compared to urban areas [9]. Nonetheless, the reasons for these observed nuances are not fully understood.

Although rural–urban status is often viewed and conceptualized for analysis as a dichotomy, it is more accurately understood as a continuum [10], with important implications for population health [10, 11]. Dichotomous measures of rural–urban status may be easier to interpret; however, important nuances between less urban and rural areas may be missed, especially in those areas of intermediate rural–urban status. There are a number of rural–urban status measures available on the national scale that attempt to capture a more detailed gradation of what it means for a geographic area (e.g., neighborhood, county, state, etc.) to be rural or urban [12].

Furthermore, the magnitude and direction of associations between income inequality and health depend on the geographic unit of analysis [13]. The association between higher income inequality and poor health outcomes is well established [1–4, 7, 9]. However, the majority of evidence demonstrating an association between income inequality and worse population health was conducted on large geographic scales, such as the national, state, or regional levels [14]. On a finer geographic scale, such as the neighborhood or municipal level, the processes related to inequality may operate differently [15]. It has been posited that income inequality at smaller scales may be less likely to reflect the degree of social stratification and endogenous inequality in the wider society, and as a result, be related to population health outcomes [14]. A 2015 study conducted in Switzerland found that mortality was actually lower in neighborhoods with high-income inequality than those with lower income inequality. This finding has been deemed the “Swiss paradox” [16].

Studies examining income inequalities and health on a fine geographic scale are lacking in the USA, however. Despite this, there is growing evidence that neighborhood and community-level factors play a unique and critical role in population health outcomes. According to an analysis by Woolf and colleagues, a “web of conditions” on the neighborhood or community level that may be difficult to disentangle contributes to individual behaviors and, therefore, population health and health inequalities [17]. These include, but are not limited to, race/ethnicity, education, socioeconomic status, the built environment, access to critical resources of everyday living (e.g., healthy foods, recreation, etc.), and social support and cohesion. Recent evidence supports the importance of examining population health inequalities on a fine geographic scale. A 2020 analysis found several key associations between population health outcomes and measures of healthcare access and social determinants of health that would have been masked had the analysis been conducted on a higher level of geospatial aggregation, such as the county or state level [18]. Another study identified small-scale associations between neighborhood-level socioeconomic status and premature mortality [19] and HIV status [20], associations which may have been masked had a higher level of spatial aggregation been used. Therefore, there is a support for and a need to investigate more nuanced relationships between socioeconomic status, inequality, community type, and health outcomes on a fine geographic scale.

To facilitate such investigations, improvements to the quality of methods and availability of national data on LE on a fine geographic scale, such as the census tract, allow researchers and policymakers to better understand the subtle but important differences of the impact of potential small-scale geographic and place-based characteristics on population health. However, to date, few studies have directly examined the potential for other socioeconomic and demographic factors to moderate or attenuate the established association between income inequality and LE. Therefore, the objective of this empirical study was to determine how income and rural–urban status potentially moderate the associations between LE and income inequality on a fine geographic scale (i.e., census tract).

## Methods

### Measures

LE at birth (2010–2015) for each US census tract was the main outcome variable and was obtained from the CDC Wonder database [21]. Census tracts are small subdivisions of a county or statistically equivalent entity, such as a city [22] and can be considered as a geographic cluster of neighborhoods or small communities. Each census tract generally contains between 1200 and 8000 people, with an optimum size of 4000 [22].

LE was then linked by census tract to socioeconomic and demographic data from the 2010 US Census [23] and the 2010 American Community Survey (ACS) [24]. The main explanatory variable was Gini index, which measures the extent to which the income distribution among units within an area deviates from a perfectly equal distribution and ranges from 0 for perfectly equitable distributions to 1 where all income is concentrated in one individual [25].

Key moderator variables from the 2010 ACS included median household income and population density in each census tract. Population density is a continuous measure and one of the most commonly used proxy measures for rural–urban status in the population health literature. All three variables—Gini index, median household income, and population density—were converted into quintiles (Q1–Q5) to capture the continuous nature of each element and to aid in interpretation [16, 26–28]. Covariates used in the analysis were percent of the population that identified as Black or African American (% Black), percent identifying as Hispanic or Latino/*a*/*x* (% Latino/*a*/x), percent with a bachelor’s degree or higher, and median age of the population in each Census tract.

Descriptive statistics (means, standard deviations, medians, and interquartile ranges [IQR]) were obtained for all study variables. Checks of normality on all study variables were conducted using Kolmogorov–Smirnov statistics and visually using *Q*–*Q* plots. Bivariate associations were estimated for all pairs of study variables (e.g., LE and Gini index) using Spearman’s rank correlation tests for non-normally distributed measures. Partial correlations were used to estimate the adjusted bivariate associations between LE and Gini index, adjusting for covariates. These partial correlations were conducted for all census tracts combined and stratified by quintile of income and rural–urban status to assess differences and linear trends in the magnitude of the associations between LE and Gini index by these factors. Average LE was also calculated for groups of census tracts cross-classified by quintile of income, rural–urban status, and Gini index. Lastly, multivariable models were used within each quintile of income inequality to examine the adjusted associations between median household income, population density, and their interaction with census-tract LE. All data were aggregated, and no individual-level data were used for this analysis. SAS version 9.4 (Cary, NC) and IBM SPSS version 27 (Armonk, NY) were used for analyses.

## Results

There were 66,857 census tracts in the analytic sample. The mean tract-level LE was 78.3 years with a standard deviation (SD) of 4.0 years, a minimum LE of 56.3 (tract # 40001376900 in Adair County, Oklahoma) and a maximum of 97.5 (tract # 37037020104 in Chatham County, North Carolina) (Table 1). The mean tract-level values for median household income were $66,792 (SD $32,688) and 0.426 (0.064) for Gini index. The mean tract-level population density was 5231 people/square mile, with a standard deviation of 11,729.

**Table 1:** Descriptive statistics for US Census tracts (*n* = 66,857)

Measure	Mean (SD)	Min–Max	Skewness	Kurtosis
Life expectancy (LE) at birth	78.3 (4.0)	56.3–97.5	− 0.26	0.36
Population density (per square mile)	5231 (11,729)	0.01–508,697	7.27	100.8
Median household income ($)	66,792 (32,688)	2744–250,000	1.41	2.86
Gini index	0.426 (0.064)	0–1	0.54	1.32
Percent Black or African American	13.9 (21.8)	0–100	2.24	4.43
Percent Hispanic/Latino/a/x	16.0 (22.8)	0–100	2.07	3.71
Median age (years)	38.1 (7.5)	12.1–94.0	0.46	1.71
Percent with bachelor’s degree or higher	30.7 (19.3)	0–100	0.88	0.09
Population size	4445 (2349)	22–72,041	2.67	29.3

Spearman correlation coefficients between LE and Gini index, median household income, percent Black population, and percent with a bachelor’s degree or higher were − 0.132, 0.672, − 0.364, and 0.608, respectively (Table 2). All were statistically significant (*p* < 0.001). Furthermore, LE was significantly correlated with population density (Spearman’s *ρ* = 0.035, *p* < 0.001). Gini index was significantly correlated with median household income (Spearman’s *ρ* = -0.329, *p* < 0.001), % Black population (Spearman’s *ρ* = 0.108, *p* < 0.001), and population density (Spearman’s *ρ* = 0.054, *p* = 0.001).

**Table 2:** Spearman’s rank correlations (and p values) among major study variables by census tract

	PD	MHHI	GINI	PBL	PHL	AGE	BACH
LE	0.035 (< 0.001)	0.672 (< 0.001)	− 0.132 (< 0.001)	− 0.364 (< 0.001)	0.065 (< 0.001)	0.236 (< 0.001)	0.608 (< 0.001)
PD		0.002 (0.669)	0.054 (< 0.001)	0.330 (< 0.001)	0.442 (< 0.001)	− 0.384 (< 0.001)	0.160 (< 0.001)
MHHI			− 0.329 (< 0.001)	− 0.309 (< 0.001)	− 0.030 (< 0.001)	0.264 (< 0.001)	0.728 (< 0.001)
GINI				0.108 (< 0.001)	− 0.050 (< 0.001)	0.057 (< 0.001)	0.004 (0.296)
PBL					0.129 (< 0.001)	− 0.350 (< 0.001)	− 0.155 (< 0.001)
PHL						− 0.415 (< 0.001)	− 0.091 (< 0.001)
AGE							0.215 (< 0.001)

*LE* life expectancy at birth, *PD* population density, *MHHI* median household income ($), *GINI* Gini index, *PBL* Percent Black or African American, *PHL* Percent Hispanic or Latino/a/x, *AGE* Median age, *BACH* Percent of population aged 25 + with at least a bachelor’s degree

**Fig. 1:**
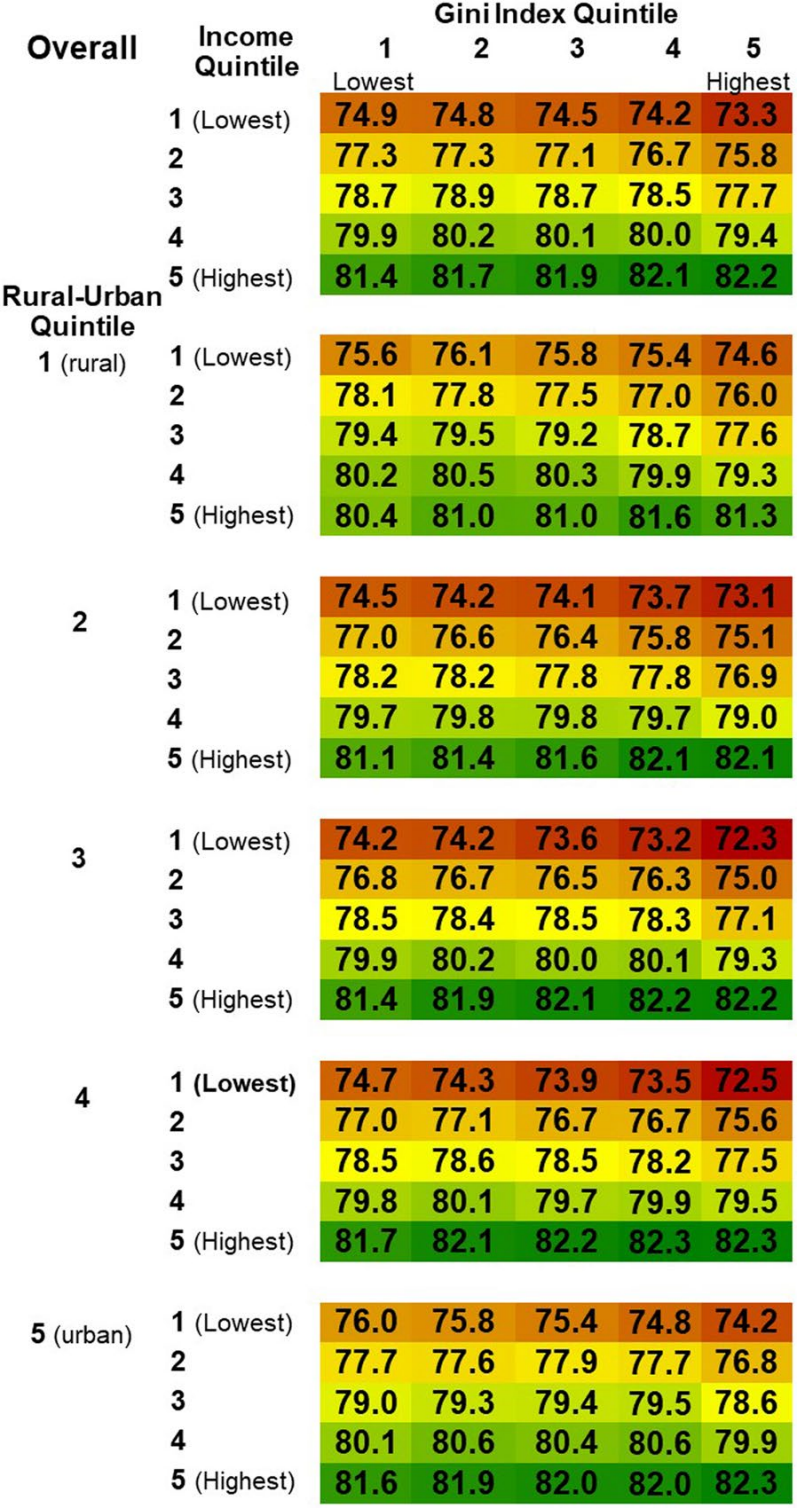
Mean census-tract life expectancy overall and cross-classified by quintile of median household income, Gini index, and rural–urban status

Substantial differences in the associations between LE and Gini index by quintile of median household income were evident (Fig. 1). Among the tracts in the lowest income quintile (Q1), LE was negatively associated with Gini index and LE decreased monotonically from 74.9 years in the tracts with the lowest Gini index to 73.3 years in the tracts with the highest Gini index (*p* = 0.003). However, among the tracts in the highest income quintile (Q5), the association between LE and Gini index was reversed and positive: higher LE was observed in areas with higher Gini indices (LE 81.4 in Q1 of Gini and 82.2 in Q2 of Gini, *p* = 0.011).

Additional variability was observed when stratifying the tracts further by rural–urban status. The lowest average LE (72.3 years) was observed in the tracts with the lowest income, highest Gini index, and with intermediate (Q3) rural–urban status. The highest average LE (82.3 years) was observed in the tracts with the highest income (Q5), second-highest Gini index (Q4) and second most urban (Q4). The monotonic trends with increasing LE with decreasing Gini index in the tracts with the lowest income generally held for all levels of rural–urban status, but the reverse trends (increasing LE with increasing Gini index) in the highest income tracts varied by rural–urban status.

**Fig. 2:**
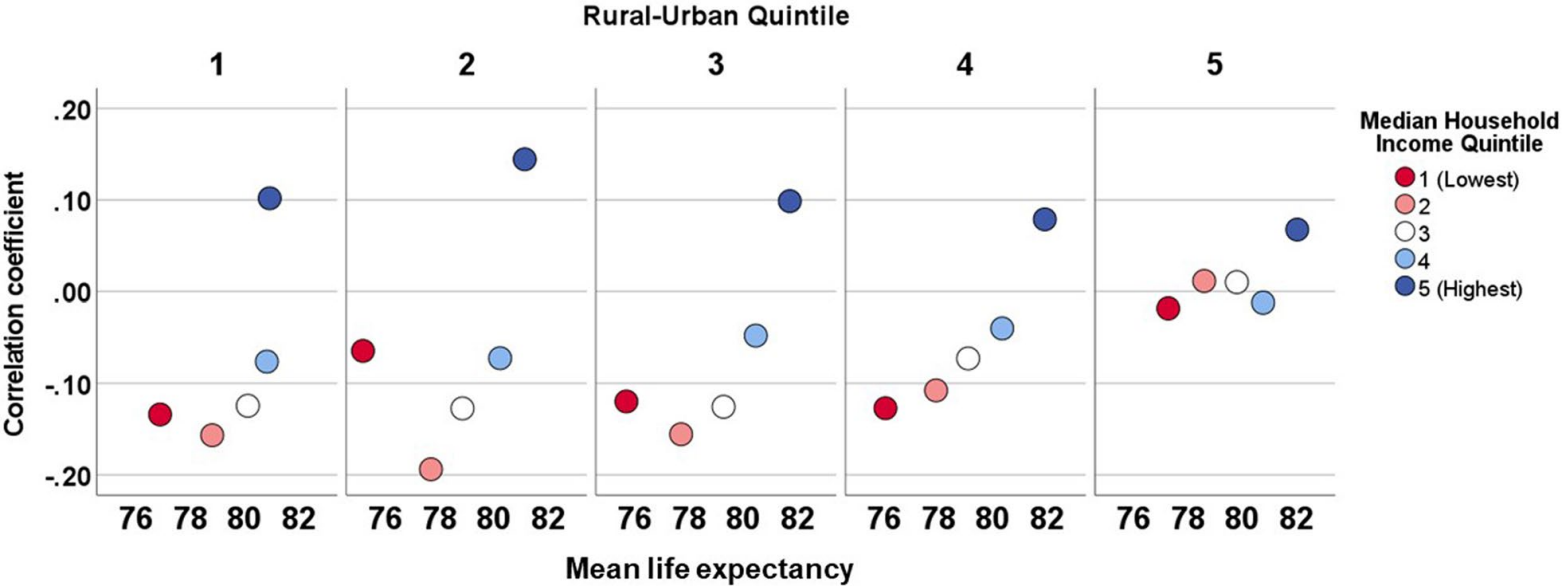
Partial correlation coefficient between Gini index and life expectancy and mean life expectancy by quintile of median household income and rural–urban status

The complex associations between Gini index and LE are further illustrated in Fig. 2. This figure shows the partial correlation coefficients between LE and Gini index in each group of census tracts, cross-classified by rural–urban status quintile and median household income quintile, as well as average LE in each class of census tracts. For the four lowest quintiles of income (Q1–Q4) in the four most rural quintiles of census tracts, the associations between LE and Gini index were significant and negative (Spearman’s *ρ* between − 0.198 and − 0.041, *p* between < 0.001 and 0.021). In contrast, the associations between LE and Gini index were significant and positive for the census tracts in the highest income quintiles, regardless of rural–urban status. For the most urban census tracts, those in the lowest quintiles of income (Q1–Q4) there were no significant associations between LE and Gini index.

Lastly, multivariable models were used to assess the associations between LE and rural–urban status and median household income, overall and by quintile of Gini index (Table 3). Overall, each one-quintile increase in population density was associated with an average decrease in life expectancy of 0.13 years (95% CI 0.11, 0.15). The association between population density and LE remained significant across all quintiles of Gini index, yet the magnitude of the association was significantly stronger in areas of low inequality than in high inequality (*p* value for trend 0.022). For median household income quintile, each one-quintile increase in income was associated with an average 1. 11-years increase in life expectancy and did not vary significantly by Gini index. The interaction terms for quintiles of rural–urban status and median household income were significant overall and for the areas with the lowest Gini index (Q1-Q3), but not significant in the census tracts with higher levels of the Gini index (Q4 and Q5).

**Table 3:** Adjusted* model estimates of main effects and interactions on census tract-level life expectancy in years (with 95% confidence intervals) overall for all census tracts and by Gini quintile (Boldface = *p* < 0.05)

	Population density quintile**	Median household income quintile***	Population density X Median household income
*Overall*			
Main effects	− 0.13 (− 0.15, − 0.11)	1.11 (1.08, 1.13)	
With interaction term	− 0.17 (− 0.21, − 0.13)	1.06 (1.02, 1.10)	0.015 (0.004, 0.027)
*Q1 (lowest Gini)*			
Main effects	− 0.13 (− 0.17, − 0.09)	1.00 (0.95, 1.05)	
With interaction term	− 0.33 (− 0.44, − 0.23)	0.80 (0.70, 0.90)	0.061 (0.032, 0.090)
*Q2*			
Main effects	− 0.18 (− 0.22, − 0.14)	1.08 (1.03, 1.13)	
With interaction term	− 0.32 (− 0.41, − 0.23)	0.95 (0.85, 1.04)	0.045 (0.019, 0.072)
*Q3*			
Main effects	− 0.18 (− 0.22, − 0.14)	1.10 (1.05, 1.16)	
With interaction term	− 0.27 (− 0.35, − 0.18)	1.02 (0.93, 1.11)	0.029 (0.004, 0.055)
*Q4*			
Main effects	− 0.12 (− 0.16, − 0.07)	1.12 (1.07, 1.18)	
With interaction term	− 0.13 (− 0.21, − 0.05)	1.11 (1.02, 1.20)	0.005 (− 0.020, 0.030)
*Q5 (highest Gini)*			
Main effects	− 0.07 (− 0.12, − 0.03)	1.00 (0.94, 1.06)	
With interaction term	− 0.01 (− 0.09, 0.08)	1.07 (0.97, 1.17)	− 0.023 (− 0.049, 0.002)

*Adjusted for tract-level percent Black or African American, percent Hispanic or Latino/a/x, median age, and percent with bachelor’s degree or higher

**p value for trend (in main effects models) < 0.05

***p value for trend (in main effects models) < 0.05

## Discussion

In this study, the association between LE and Gini index in most US census tracts was negative, supporting the vast literature concluding that lower income inequality is associated with better population health [1–7]. However, those associations were not present in more urban areas. Furthermore, in the census tracts with the highest income, the association was reversed: higher LE was associated with higher Gini indices. These findings are partially antithetical to the “income inequality thesis”, which states that increasing wealth is associated with improved population health, but only to a certain level of wealth [14]. Once this threshold of wealth is reached, reducing income inequality is the most important driver of improving population health [15]. Study findings support the tenet of the income inequality thesis proposing that increasing wealth is associated with better population health, as measured through LE. Increasing median household income was associated with increased tract-level LE. However, the findings contradict the part of the income inequality thesis that for sufficiently high levels of wealth, reducing income inequality is associated with increased LE. For the wealthiest census tracts, increasing LE was associated with increased income inequality, consistent with the Swiss study described earlier [16]. However, more research is needed to determine if these unexpected associations are observed for other population health outcomes [29, 30].

The findings for rural–urban status and LE were more complex. The association between income inequality and LE was strongest in areas outside the most rural (Q1) and most urban (Q5) census tracts. The reasons for this are not well understood. One explanation for this finding is that areas with greater poverty could be more vulnerable to the deleterious effects of income inequality on health, especially when those areas lie in the intermediate areas of rural–urban status. Broadly speaking, while not specific to urban areas, over the past century, urban areas are more likely to have adequate housing, access to primary health care, sanitation, and resources [31], which may temper the associations between LE and inequality, even in lower income areas. Likewise, there are potential health benefits to living in highly rural and remote areas, such as the lower cost of living, access to green space, pace of life, improved environmental factors (e.g., pollution) [32–34]. However, like the benefits of urban living, these attributes are not unique to the most rural and remote areas. Therefore, the reasons for these highly nuanced differences in the strength and direction of association between LE and income inequality jointly by wealth and rural–urban status remain unclear and merit further research.

The empirical findings of the current study should be interpreted in the context of several limitations. First, estimating LE on a small geographic scale such as census tract is subject to systematic errors [35]. Second, the partial correlations only adjusted for two factors, percent Black population and percent with a bachelor’s degree or higher, and therefore the observed associations are subject to residual confounding. This analysis only considered moderation of the association between income inequality and LE by two factors, income and rural–urban status. Factors, such as race/ethnicity, education, and built environment, likely moderate these associations. The analysis also did not consider potential regional differences in the association between income inequality and LE [36]. Only one measure of each main predictor variable was used, largely due to limited variables available at the census tract level. Patterns of association and moderation may vary based on which measure of health was used [37]. This study used Gini index as the main measure of income inequality [38]. As with all measures of income inequality, the Gini index is only somewhat sensitive to income inequality occurring in the middle of the income distribution [39]. Future studies could determine if the observed associations are sensitive to the type of income inequality measure used, such as the Atkinson Index, which is less sensitive to differences in the middle of the distribution. Also, the analysis considered only one measure of rural–urban status—population density. As there is no universally accepted and utilized measure of rural–urban status, it is possible that the observed associations would also change if a different measure of rural–urban status, such as distance to the nearest metropolitan area or percent urban population, were used [40]. Lastly, for analysis and interpretation, continuous measures—area-level income, income inequality, and population density—were categorized into quintiles. Choosing other cut-points (i.e., quartiles) may result in different patterns of associations.

Despite these limitations, this study provides empirical evidence that the widely established principle that areas with lower income inequality generally experience better population health may not extend to all areas and, in fact, may be reversed among high-income populations. The rationale for these findings is unclear and requires further research. While this analysis found that for most census tracts, the established associations held, the variation by income suggests that any efforts to improve population health through reducing income inequality must be tailored to the needs of distinct populations to maximize effectiveness. Future research should focus on identifying and addressing these nuanced associations that lead to critical health inequalities that may be masked when examining such associations on a higher geographic level of aggregation [41].

## Data Availability

The datasets used and/or analyzed during the current study are available from the corresponding author upon reasonable request.
